# Neddylation Regulates Macrophages and Implications for Cancer Therapy

**DOI:** 10.3389/fcell.2021.681186

**Published:** 2021-06-07

**Authors:** Yanyu Jiang, Lihui Li, Yan Li, Guangwei Liu, Robert M. Hoffman, Lijun Jia

**Affiliations:** ^1^Longhua Hospital, Cancer Institute, Shanghai University of Traditional Chinese Medicine, Shanghai, China; ^2^College of Life Sciences, Beijing Normal University, Beijing, China; ^3^Department of Surgery, University of California, San Diego, San Diego, CA, United States; ^4^AntiCancer Inc., San Diego, CA, United States

**Keywords:** macrophage, neddylation, cytokine, migration, polarization

## Abstract

Tumor-associated macrophages (TAMs) promote cancer progression via stimulating angiogenesis, invasion/metastasis, and suppressing anti-cancer immunity. Targeting TAMs is a potential promising cancer therapeutic strategy. Neddylation adds the ubiquitin-like protein NEDD8 to substrates, and thereby regulates diverse biological processes in multiple cell types, including macrophages. By controlling cellular responses, the neddylation pathway regulates the function, migration, survival, and polarization of macrophages. In the present review we summarized how the neddylation pathway modulates Macrophages and its implications for cancer therapy.

## Introduction

The tumor microenvironment (TME) comprises multiple cell types, including tumor cells, endothelial cells, fibroblasts and immune cells, interacting with each other continuously (Junttila and de Sauvage, 2013). TME is the critical mediator to inhibit or promote tumor progression and metastasis (Junttila and de Sauvage, 2013). Macrophages are the most abundant immune-cell population in TME (Qian et al., 2011; Izumi et al., 2013; Vakilian et al., 2017). Macrophages can produce various cytokines, such as interleukin-6 (IL-6), tumor necrosis factor-α (TNF-α), interferon gamma (IFN-γ), which are inflammatory factors (Mantovani, 2010; Cassetta and Pollard, 2018). In turn, chronic inflammation promotes macrophages infiltration to initiate tumor growth via inducing gene mutations and resistance to apoptosis (Coussens and Werb, 2002; Shacter and Weitzman, 2002; Nagarsheth et al., 2017). In established tumors, macrophages stimulate tumor growth, migration, angiogenesis, and metastasis via the following mechanisms: (1) Macrophages create and maintain the tumor vascular network by producing and releasing pro-angiogenic cytokines, such as vascular endothelial growth factor-α (VEGF-α) and angiogenic CXC chemokines (CXCL8 and CXCL12), transforming growth factor-β (TGF-β) and TNF-α (Noy and Pollard, 2014; Cassetta and Pollard, 2018). (2) Macrophages produce pro-invasive extracellular matrix-degrading proteases, such as matrix metalloproteinase 9 (MMP9), to promote cancer cell intravasation and metastasis (Pollard, 2004; Noy and Pollard, 2014; Jinushi and Komohara, 2015; Cassetta and Pollard, 2018). (3) Macrophages serve as an important immunosuppressive regulator to avoid cancer-cell eradication via suppressing T-cell development, activation or function (Cassetta and Pollard, 2018; DeNardo and Ruffell, 2019). Elevated macrophage infiltration in tumors is associated with higher tumor grade and worse overall survival in diverse forms of cancers, such as breast cancer, lung cancer, and lymphoma (Steidl et al., 2010; Zhao et al., 2017; Zhang et al., 2018). An increase of macrophages in tumors suppresses tumor response to first-line therapy, such as irradiation, chemotherapy, immunotherapy (Ruffell and Coussens, 2015; Petty and Yang, 2017; DeNardo and Ruffell, 2019). A decrease of macrophages in the TME correlates with decreased tumor growth/metastasis and increased survival (Mantovani et al., 2017). Thus, macrophages are a promising target for cancer therapy.

Currently, macrophages are targets in some cancer therapy, including: (1) Depletion macrophages via targeting colony-stimulating factor 1 (CSF1) and colony-stimulating factor 1 receptor (CSF1R) pathway, such as with the small molecule PLX3397 (Butowski et al., 2016; Yan et al., 2017). (2) Promoting macrophage death or inhibiting macrophage proliferation in TME with bisphosphonates (Stresing et al., 2007). (3) Inhibition macrophage infiltration in the TME by targeting the C-C motif chemokine ligand 2 (CCL2) and C-C motif chemokine receptor 2 (CCR2) axis with Carlumab (Loberg et al., 2007). (4) Reprogramming macrophages via anti-CD47 or CD40 antibodies to activate the antitumor activity (Cassetta and Pollard, 2018). These macrophage-targeted therapeutic approaches have shown promise in preclinical models and are being investigated in Phase I/II clinical trials as monotherapy or in combination with chemotherapy or radiation (Cassetta and Pollard, 2018).

**FIGURE 1:**
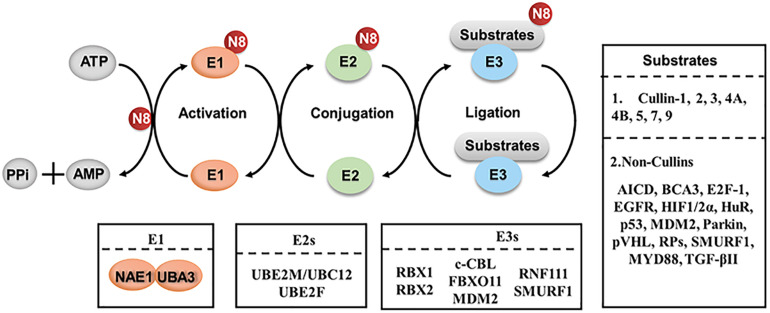
The process of protein neddylation. Neddylation is a biochemical process of adding an ubiquitin-like protein, NEDD8, to substrates via a three-step enzymatic cascade involving NEDD8-activating enzyme El, NEDD8-conjuagating enzyme E2 and substrate specific NEDD8-E3 ligases. N8, NEDD8.

Recently, neddylation also has emerged as a critical mechanism in regulating macrophages. Neddylation, a type of post-translational modification, is a biochemical process of adding an ubiquitin-like protein NEDD8 (neuronal precursor cell-expressed developmentally down-regulated protein 8) to substrates via a three-step enzymatic cascades (Kamitani et al., 1997; Xirodimas, 2008; Enchev et al., 2015). Similar to ubiquitination, NEDD8 is first activated by an E1 enzyme (NEDD8 activating enzyme, NAE), transferred to an E2 enzyme (Ubc12/UBE2M and UBE2F), and then conjugated to substrates via a specific E3 enzyme (such as RBX1, RBX2) (Gong and Yeh, 1999; Walden et al., 2003; Huang et al., 2005; Zhao et al., 2014; Enchev et al., 2015; Zhou et al., 2018; Figure 1). Neddylation modification regulates diverse biological processes via affecting the stability, conformation, localization and function of its substrate proteins (Zhao et al., 2014; Enchev et al., 2015). The best-characterized physiological substrates of neddylation pathway are the cullin subunits of Cullin-RING ligases (CRLs) (Zhao and Sun, 2013). As the largest family of E3 ubiquitin ligases, CRLs promote the ubiquitination and degradation of approximately 20% of cellular proteins via the ubiquitin-proteasome system (Petroski and Deshaies, 2005; Nakayama and Nakayama, 2006; Deshaies and Joazeiro, 2009; Soucy et al., 2009). Neddylation modification to the C-terminal lysine residue of cullin changes the conformation of CRLs and activates CRLs enzymatic function for protein ubiquitination and degradation (Jia and Sun, 2011; Chen et al., 2016).

Recent studies from our and other groups demonstrate that protein neddylation (NEDD8 and NEDD8-conjugated proteins) and the key components of the neddylation pathway (NAE, UBE2F, UBE2M, RBX1, RBX2) are overactivated in multiple human cancers (Li et al., 2014; Hua et al., 2015; Xie et al., 2017; Zhou et al., 2017; Yu et al., 2018; Tian et al., 2019; Jiang et al., 2020; Wang et al., 2020). The overactivated neddylation pathway activates CRLs to degrade many tumor-suppressor proteins, such as p21and p27, leading to tumorigenesis and tumor progression, and resulting in a worse overall patient survival (Li et al., 2014, 2019; Zhou et al., 2018; Jiang et al., 2020).

In 2009, a specific small molecular inhibitor of NAE, called MLN4924 (also known as pevonedistat), was identified via high throughput screening (Soucy et al., 2009). MLN4924 forms a covalent NEDD8-MLN4924 adduct at the active site of NAE to inhibit the first step of the neddylation enzymatic process (Brownell et al., 2010; Enchev et al., 2015). By doing so, MLN4924 inhibits the entire neddylation pathway and blocks the activation of CRLs, thus inducing the accumulation of various tumor-suppressive CRL substrates which trigger cell-cycle arrest, DNA damage, apoptosis or senescence (Zhou et al., 2018; Liang et al., 2020; Zhou and Jia, 2020). Phase II/III clinical trials of MLN4924 have been conducted for the treatment of several solid tumors and hematologic malignancies (Swords et al., 2015, 2018; Bhatia et al., 2016; Sarantopoulos et al., 2016; Shah et al., 2016).

The neddylation pathway also modulates macrophages and their response to different stimulation (Li et al., 2013; Zhou et al., 2019a, b), thus highlighting the connection between neddylation, macrophages, and cancer.

## Neddylation Regulates the Release of Inflammatory Cytokines in Macrophages

**FIGURE 2:**
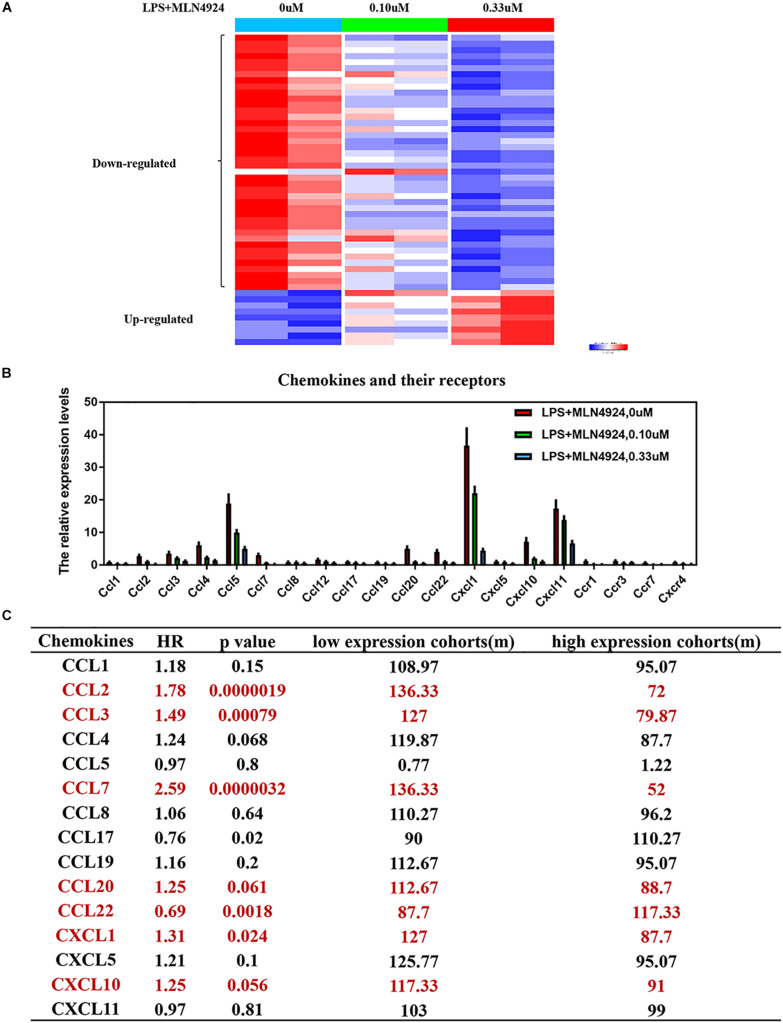
Neddylation regulates the release of inflammatory cytokines in macrophages. **(A)** The results of PCR array analysis on lipopolysaccharide (LPS)-treated RAW264.2 upon MLN4924 treatment, **(B)** MLN4924 treatment decreases the level of chemokines and the related receptors in lipopolysaccharide (LPS)-treated RAW264.2. **(C)** The survival analysis of these chemokines in lung adenocarcinoma using KM plotter website.

Inflammatory cytokines secreted by macrophages are small, secreted proteins that regulate immune-cell development, recruitment and trafficking and are potential targets for cancer therapy (Robinson et al., 2002; Tsuyada et al., 2012; Nagarsheth et al., 2017; Liu et al., 2020). Inactivation the neddylation pathway suppresses proinflammatory cytokine production by macrophages (Chang et al., 2012; Li et al., 2013; Asare et al., 2017). For example, inactivation of neddylation with MLN4924 in macrophages inhibits LPS-induced inflammatory cytokine production, such as IL-6, TNF-α, and IL-1β (Chang et al., 2012; Li et al., 2013; Asare et al., 2017). RBX2-overexpressing macrophages upregulate pro-tumorigenic cytokines (IL-6, TNF-α, and IL-1β), and downregulate anti-tumorigenic cytokine (IL-12) and anti-inflammatory cytokine (IL-10) (Chang and Ding, 2014). In addition, proteasome inhibitors (e.g., MG-132) repress LPS-induced up-regulation of certain proinflammatory cytokines, such as IL-6, TNF-α, and IL-1β (Ortiz-Lazareno et al., 2008). Furthermore, our group found that neddylation regulates macrophage production of several cytokines (Figure 2A). PCR array analysis on MLN4924-treated RAW264.2 demonstrated that the levels of 51 inflammation-related factors were altered (42 down-regulated and 9 up-regulated) compared to lipopolysaccharide (LPS) treaded RAW264.2 (Figure 2A). Among these factors, the classical inflammatory factors, including IL-6, IL-18, TNF-α, IFN-γ, IL-1α, IL-1β, and CRP (C-reactive protein) were significantly decreased (Figure 2A).

Apart from cytokines, our group found that chemokine (C-C motif) ligand families (CCL-1,2,3,4,5,7,8,12,17,19,20,22), chemokine (C-X-C motif) ligand families (CXCL1,5,10,11) and the related receptors (CCR1, CCR3, CCR7, and CXCR4) were significantly decreased upon MLN4924 treatment in lipopolysaccharide (LPS)-treated RAW264.2 (Figure 2B). Among these chemokines, high expression of CCL2, CCL3, CCL7, CCL20, CXCL1, and CXCL10, is correlated with poorer overall survival of cancer patients than patients with low expression (*p* < 0.05) (Gyorffy et al., 2013, 2014; Menyhart et al., 2018; Nagy et al., 2018; Figure 2C). CCL2 promotes the infiltration of monocytes, thus promoting cancer-cell vascularization, extravasation and metastasis (Fridlender et al., 2011; Qian et al., 2011; Tsuyada et al., 2012; Wolf et al., 2012; Bonapace et al., 2014; Li et al., 2017). CCL3 promotes tumor extravasation (Robinson et al., 2002; Farmaki et al., 2017). CXCL1 is overexpressed in tumors and recruits the infiltration of monocytes to promote tumor progression, chemoresistance, and metastasis (Acharyya et al., 2012; Miyake et al., 2016; Wang et al., 2016, 2017, 2018; Hsu et al., 2018; Yang et al., 2019). Overactivated neddylation may contribute to tumor progression via promoting the macrophages-mediated inflammation response, but more detailed characterizations and effects are still warranted.

## Neddylation Regulates Inflammation-Related Signal Pathways in Macrophages

**FIGURE 3:**
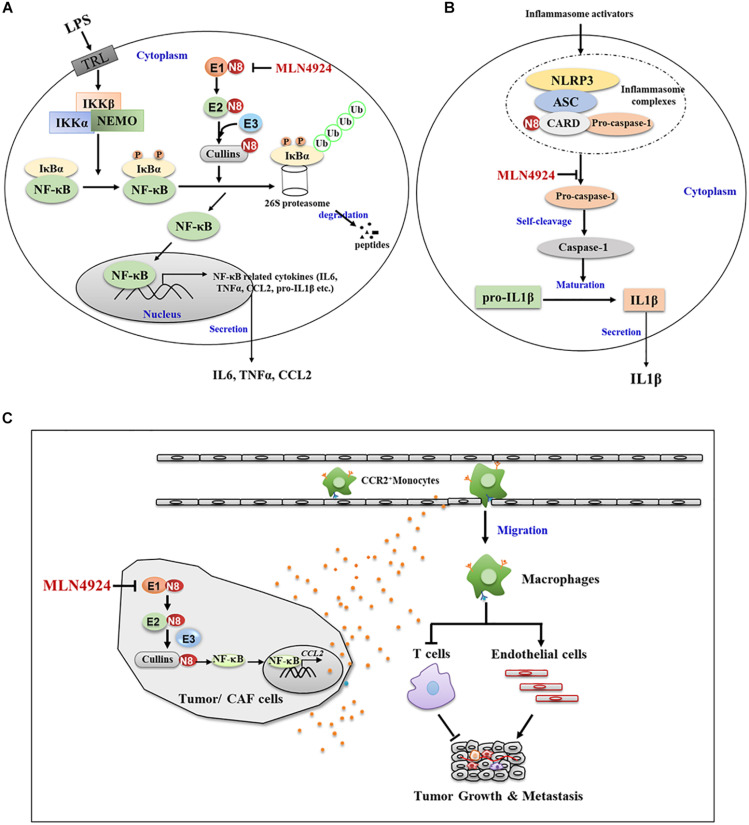
Neddylation regulates the function and migration of macrophages. **(A)** Neddylation inactivation by MLN4924 inhibits the activity of CRLs and induces the accumulation of its substrate IKBα, which sequesters NF-KB in the cytoplasm to block NF-KB transcriptional activity. **(B)** MLN4924 inhibits neddylation modification of caspase-1 CARD domain, and thus diminishes caspase-1 maturation and reduces IL-1β maturation and secretion. **(C)** Inactivation neddylation in tumor or CAF cells inhibited CCL2 expression and macrophage infiltration, thus mediating its lung metastasis-inhibitory efficacy.

Transcription factors are intracellular molecules that modulate the activity of specific genes. When macrophages are stimulated, transcription factors activate related genes to eliminate pathogens or other dangerous elements. Nuclear factor kappa-B (NF-κB), one of the basic inflammatory-related factors, functions as a precursor to increase the concentration of proinflammatory factors and thus coordinates the inflammatory response (DiDonato et al., 2012). In normal conditions, NF-κB is sequestered in the cytoplasm by interacting with its inhibitory protein IκBα (Bhoj and Chen, 2009). When stimulated by various signals, neddylation modification to the C-terminal lysine residue of cullin changes the conformation of CRLs and activates CRLs enzymatic function for IκBα ubiquitination and degradation (Bhoj and Chen, 2009; Chang et al., 2012; Jin et al., 2018). The degradation of IκBα by the ubiquitin proteasome system allows NF-κB entering into nucleus where it binds to DNA promoter regions, thus turning on transcription of a wide spectrum of genes and the release of inflammatory factors (Bhoj and Chen, 2009). This process is triggered by IκBα kinases (IKKα or β), which phosphorylate IκBα at S32 and S36 (Fuchs et al., 1999; Tan et al., 1999), thus, highlighting the underlying cooperative relationship between phosphorylation and neddylation whereas. Inactivation of neddylation inhibits the activity of CRLs and induces the accumulation of its substrate IκBα, which sequesters NF-κB in the cytoplasm to block NF-κB transcriptional activity (Chang et al., 2012; Jin et al., 2018; Figure 3A). Moreover, Cullin 5 neddylation following LPS stimulation triggers the interaction with tumor necrosis factor receptor-associated factor 6 (TRAF6), an essential adaptor to promote the activation of NF-κB, thus inducing K63-linked TFAR6 polyubiquitination and leading to NF-κB activation, and eventually facilitating the generation of proinflammatory cytokines (Zhu et al., 2016, 2017).

Apart from the modulation of transcription factors, the neddylation pathway regulates the maturation and secretion processes of inflammatory factors in macrophages. For example, the association of pro-caspase-1 with NLR family pyrin domain containing 3 (NLRP3)/apoptosis-associated speck-like (ASC) protein via caspase recruitment domain (CARD) promotes the autocatalytic activity of pro-caspase-1 to self-cleavage into caspase-1, and thus leads to the maturation of 31 KD pro-interleukin-1β into 17KD IL-1β (Bryant and Fitzgerald, 2009; Dowling and O’Neill, 2012). In this process, neddylation modification to the CARD domain is required for the self-cleavage of pro-caspase-1 to generate its catalytically active subunits (Segovia et al., 2015). NEDD8 silencing or MLN4924 inhibition of neddylation modification of the caspase-1 CARD domain diminishes caspase-1 maturation and inhibits IL-1β maturation and secretion (Segovia et al., 2015; Figure 3B). These findings demonstrate how neddylation pathway modulates the macrophage inflammation response, which provides a molecular basis for targeting neddylation pathway in macrophages to ameliorate the inflammation microenvironment in tumors.

## Neddylation Regulates the Migration of Macrophages

The monocyte-derived macrophages are mainly recruited into tumors by chemokines, which can be released from cancer cells or stromal cells (Qian et al., 2011; Izumi et al., 2013; Vakilian et al., 2017). Among these chemokines, CCL2 recruits monocytes into tumors (Qian et al., 2011; Izumi et al., 2013; Vakilian et al., 2017). High CCL2 expression positively correlates with increased infiltration of tumor associated macrophages and predicts worse prognosis in multiple human and murine cancers (Fridlender et al., 2011; Qian et al., 2011; Tsuyada et al., 2012; Wolf et al., 2012; Bonapace et al., 2014; Li et al., 2017).

A recent study from our group showed that the elevated neddylation pathway in cancer cells led to the accumulation of NF-κB-regulated activation of chemokines CCL2 with promotion of macrophage infiltration (Zhou et al., 2019a). Inactivation neddylation in cancer cells, either pharmacologically (MLN4924) or genetically (NEDD8 knock out via Crisp Cas9), inhibited CCL2 expression and macrophage tumor infiltration, thus inhibiting lung metastasis (Zhou et al., 2019a; Figure 3C). MLN4924 also suppressed cancer-associated fibroblasts (CAF)-derived and macrophage-derived CCL2 (Zhou et al., 2019b; Figure 3C). Therefore, neddylation activation promotes the migration of macrophages via regulating tumor/CAF-derived CCL2, indicating synergistic inhibition of neddylation in CCL2-producing cells to target the CCL2-macrophage axis. MLN4924 can thus reduce macrophage accumulation in tumors, which could be an effective cancer therapy.

Functionally, tumor infiltrated macrophages induce an immunosuppressive and tumorigenic phenotype by neutralizing the function of cytotoxic CD8^+^ T cells (Cassetta and Pollard, 2018). Neddylation inactivation decreases macrophage tumor infiltration and promotes CD8^+^ T cell tumor infiltration (Zhou et al., 2019a). Based on these findings, we postulate that targeting the neddylation pathway to inhibit macrophage recruitment in tumors would be tested in clinical trials.

## Neddylation Regulates the Proliferation and Survival of Macrophages

**FIGURE 4:**
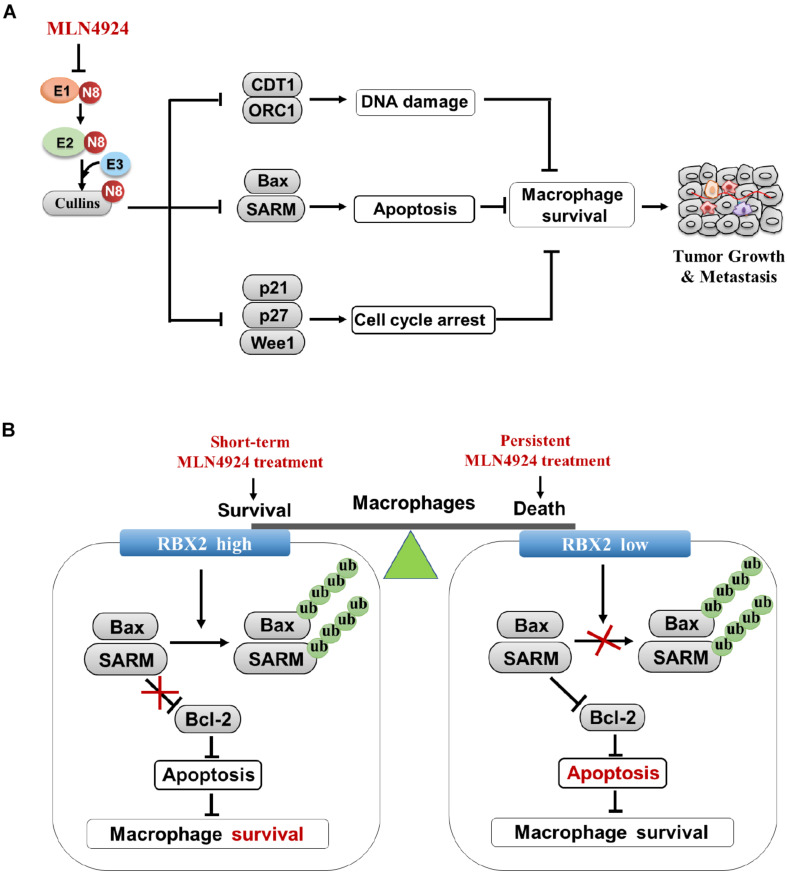
Inhibition of neddylation pathway impairs proliferation and survival of macrophages. **(A)** Multiple anti-growth mechanisms in macrophages upon MLN4924 treatment. **(B)** Neddylation modification equilibrates the survival/inflammatory response and death of macrophages.

Similar to cancer cells, the neddylation pathway is required for the proliferation and survival of macrophages. Neddylation inactivation inhibits macrophage viability with the following mechanisms, including: (1) Neddylation inactivation by MLN4924 blocks cullin neddylation and suppresses CRL activity, thus leading to the accumulation of cell-cycle inhibitors (e.g., p21, p27, and Wee1) and inducing G_2_-M- phase cell-cycle arrest in macrophages. (2) MLN4924 activates DNA re-replication stress and DNA damage by inducing the accumulation DNA replication licensing protein of CDT1 and ORC1 in macrophages. (3) MLN4924 triggers the increase of tumor-suppressive CRL substrate NF-κB inhibitor IκBα, and resulting in apoptosis of macrophages (Li et al., 2013; Zhou et al., 2019b; Figure 4A). (4) RBX2 depletion in macrophages induces the accumulation of proapoptotic Bax and SARM, and inhibits the expression of anti-apoptotic protein Bcl-2, thereby activating cytosolic cytochrome c, caspase-9 and caspase -3, and leading to macrophage’s death (Chang and Ding, 2014).

How dose neddylation modification influence survival and the inflammatory response of macrophages? Firstly, partial inhibition of neddylation by MLN4924 inhibits inflammatory response of macrophages at an early stage when cell viability is not significantly blocked. However, continuous inactivation of neddylation by MLN4924 impairs macrophage viability, indicating that the balance of macrophage survival or death depends on the treatment degree of neddylation inactivation by MLN4924 treatment (Li et al., 2013; Figure 4B). Secondly, RBX2-overexpressing macrophages maintain viability via degradation of the pro-apoptotic proteins (BAX and SARM), which facilitate the pathogen-associated molecular patterns (PAMPs)-stimulated inflammatory response. RBX2 knockdown induces the accumulation of BAX and SARM to trigger intrinsic apoptosis (Chang and Ding, 2014; Figure 4B), suggesting the RBX2-dependent ubiquitin-proteasome system serves as a checkpoint between the survival and death of macrophages. These results suggest strategies for targeting neddylation to inhibit tumor infiltration macrophages as potential cancer therapy.

## Neddylation Regulates the Polarization of Macrophages

Macrophages can be divided into classically activated macrophages (M1 phenotype) and alternatively activated macrophages (M2 phenotype) (Gordon and Taylor, 2005; Murray, 2017; Chistiakov et al., 2018). M1 produces inducible nitric oxide synthase (iNOS) and pro-inflammatory cytokines upon LPS and/or IFN-γ stimulation, which have anti-cancer effects. M2 is triggered by IL-4 or IL-13, to produce arginase 1 (Arg1) and anti-inflammatory cytokines, eventually promoting tumorgenesis (Martinez et al., 2006; Liu et al., 2014).

**FIGURE 5:**
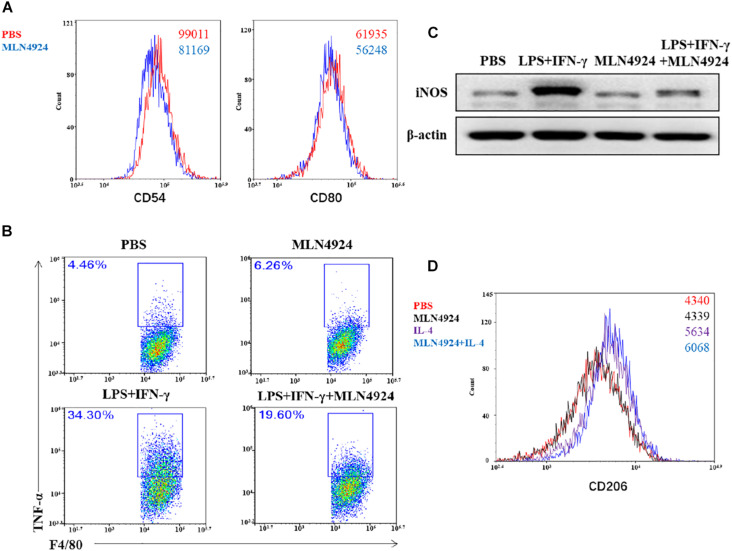
Neddylation regulates the polarization of macrophages. **(A,B,D)** Flow cytometric analysis of MLN4924-treated BMDMs, LPS and IFN-γ-induced Ml macrophages, and IL-4-induced M2 macrophages. BMDMs were isolated from 8-week old wild type mouse and stimulated with L929 supernatant for 7 days, and then treated with MLN4924 (0.5 μM for 24 h). **(C)** The expression of iNOS in BMDMs after stimulation by LPS and IFN-γ for 24 h.

Asare et al. (2017) reported that MLN4924 drove macrophages to the anti-inflammatory M2 state with increase of M2 makers, arginase-1 and IL-13, and decrease M1 markers, TNF-α, IL-6, and IL-12 in bone marrow-derived macrophages (BMDMs) which were isolated from Apoe^–/–^ knockout mice. Our team also found that MLN4924 inhibited macrophages to the M1 phenotype in wild type mouse bone-marrow derived macrophages (Figure 5). Flow cytometric analysis demonstrated that the expression level of costimulatory molecules, CD80 and CD54, was decreased in MLN4924-treated BMDMs, indicating that MLN4924 suppressed the polarization of BMDMs into M1 macrophages (Figure 5A). To further confirm this hypothesis, we treated BMDMs with MLN4924 to determine the phenotype switching between M1 and M2 macrophages. As shown, the proportion of LPS and IFN-γ-induced M1 macrophages (F4/80+TNF-α+) was significantly reduced upon MLN4924 treatment (Figure 5B). Also, after 24 h stimulation, LPS and IFN-γ induced the expression of iNOS in BMDMs, which was restored by MLN4924 treatment (Figure 5C). IL-4 stimulation resulted in the M2 phenotype (CD11b+/F4/80+/CD206+), while MLN4924 up-regulated the number of M2 macrophages following IL-4 treatment (Figure 5D), indicating that inactivation of the neddylation pathway by MLN4924 polarized macrophages toward a M2 phenotype *in vitro*. In a metastatic lung cancer model, NEDD8 knockout significantly reduced the population of both M1 (CD11b+/F4/80+/CD206-) and M2 (CD11b+/F4/80+/CD206+) macrophages, suggesting that neddylation pathway probably mainly regulates the chemotaxis of macrophages but not the polarization in the metastatic lung cancer model (Zhou et al., 2019a).

In summary, these results imply that neddylation regulates the polarization of macrophages in a cell-type and microenvironment-type dependent manner. Additional investigation is needed to further decipher the detailed mechanisms. Nevertheless, in either case, a high number of macrophages in tumor is associated with poor overall survival. Therefore, limiting the numbers of macrophages via inactivating neddylation in tumor is a promising therapeutic strategy.

## Conclusion and Remarks

Macrophages are a major component of TME. Neddylation inactivation to suppress the accumulation of macrophages in tumor is a novel and promising cancer therapeutic strategy (Zhou et al., 2019a, b). However, some questions still await further investigation.

Firstly, further studies are needed to fully identify macrophage phenotypes and define the determining factors for macrophage’s polarization upon MLN4924 treatment in various tumor models. Secondly, the TME comprises different types of infiltrated immune cells, fibroblasts, endothelial cells as well as cancer cells. The role of the neddylation genes (such as NEDD8, UBA3, NAE1, UBE2M, UBE2F, RBX1, and RBX2) in specific cell subsets of the TME needs to be further clarified. Thirdly, we need to learn how neddylation modulates the proliferation and differentiation of hematopoietic stem cells and myeloid progenitor cells, and how neddylation modulates the production of monocytes in multiple tumor models. Fourthly, the efficacy of MLN4924 in combination with cancer immunotherapy (such as nivolumab, avelumab, ipilimumab) needed to be tested. Finally, identification of the biomarkers indicating the viability of macrophages upon neddylation inhibition could maximize the therapeutic efficacy of MLN4924, and optimize the dose, routine, and schedule.

Once we assure these questions, the regulatory mechanisms of macrophages will be clearly clarified, which would extend our understanding of how neddylation pathway modulates macrophages in fundamental cancer biology, and provide a sound rationale and molecular basis for neddylation-based targeting macrophages therapies for clinical cancer treatment.

## Author Contributions

YJ, LL, and YL collected the related manuscript and drafted the manuscript. GL and RMH provided the technical or material support. LJ, YJ, and RMH revised and finalized the manuscript. All authors read and approved the final manuscript.

## Conflict of Interest

RMH was employed by company Anticancer Inc. The remaining authors declare that the research was conducted in the absence of any commercial or financial relationships that could be construed as a potential conflict of interest.
